# Degenerated primer design to amplify the heavy chain variable region from immunoglobulin cDNA

**DOI:** 10.1186/1471-2105-7-S4-S9

**Published:** 2006-12-12

**Authors:** Ying Wang, Wei Chen, Xu Li, Bing Cheng

**Affiliations:** 1 Center for Laboratory Medicine, First Teaching Hospital of Medical School, Xi'an Jiaotong University, Xi'an 710061, China; 2 Department of Biomedical Engineering, Xi'an Jiaotong University, Xi'an 710049, China

## Abstract

**Background:**

The amplification of variable regions of immunoglobulins has become a major challenge in the cloning of antibody genes, whether from hybridoma cell lines or splenic B cells. Using conventional protocols, the heavy-chain variable region genes often are not amplified successfully from the hybridoma cell lines.

**Results:**

A novel method was developed to design the degenerated primer of immunoglobulin cDNA and to amplify cDNA ends rapidly. Polymerase chain reaction protocols were performed to recognize the VH gene from the hybridoma cell line. The most highly conserved region in the middle of the VH regions of the Ig cDNA was identified, and a degenerated 5'primer was designed, using our algorithms. The VH gene was amplified by both the 3'RACE and 5'RACE. The VH sequence of CSA cells was 399 bp.

**Conclusion:**

The new protocol rescued the amplifications of the VH gene that had failed under conventional protocols. In addition, there was a notable increase in amplification specificity. Moreover, the algorithm improved the primer design efficiency and was shown to be useful both for building VH and VL gene libraries and for the cloning of unknown genes in gene families.

## Background

The amplification of variable region (Fv) of immunoglobulin (Ig) by reverse transcription polymerase chain reaction (RT-PCR) has become an invaluable technique for studying antigen-antibody interactions and cloning monoclonal antibodies (mAbs) for medical purposes [1]. All approaches require amplification or cloning of the heavy-chain variable regions (VH) and light-chain variable regions (VL) cDNAs, which are responsible for the antigen-antibody interactions and present an important diversity in their amino acid composition. The specific amplification of antibody Fv genes is a major challenge in cloning Fv genes, whether expressed in hybridoma cell lines or in a population of splenic B cells. This is due to the fact that the mouse Ig genes are highly diverse in their amino acid composition and nucleotide sequence.

When isolating VH and VL genes from hybridoma cell lines, the most widespread solution is either to use the specific consensus primers suggested to be "universal" or use the commercially available primer sets to isolate the variable (V) domains. Because 3' primer design often covers the isotype specific constant region sequences, 5' primer design is generally focused. Previous studies indicated that using the primer sets might give more chance of success than the "universal" primers [2]. However, the failure of the primer sets or the "universal" primers to amplify certain V gene segments has recently been documented by several authors. Some research has noted that only four out of ten V genes of Ig cDNAs were amplified [3].

In our study, we initially employed the "universal" primers based on Zhou et al. [4] designed for amplifying mouse V genes from three hybridoma cell lines. The VL regions were amplified successfully. However, the VH region was not amplified from one hybridoma cell line CSA. Commercially available mouse primer sets from Pharmacia Corporation designed for mouse scFv library construction were used to amplify the cell line. But the result was still unsuccessful. This prompted us to design our own primer. But most existing algorithms and programs of primer selection have a lot of shortcomings for a large gene family. Moreover, they could not balance the specificity and the number of primers. We wanted to design as small as possible a set of primers to amplify the target gene. So we developed an efficient algorithm, which could identify the most highly conserved region of Ig VH fragments, then a specific degenerated 5'primer was designed, which rescued the failed VH region followed by 3'RACE and 5'RACE PCR.

## Results

### Conventional PCR with the "universal" primers and commercially available primer sets

The specific amplification product of predicted size from the hybridoma cell line CSA was not observed using the "universal" primers or the commercial primer sets.

### RACE with the primer designed by our algorithm

**Figure 1:**
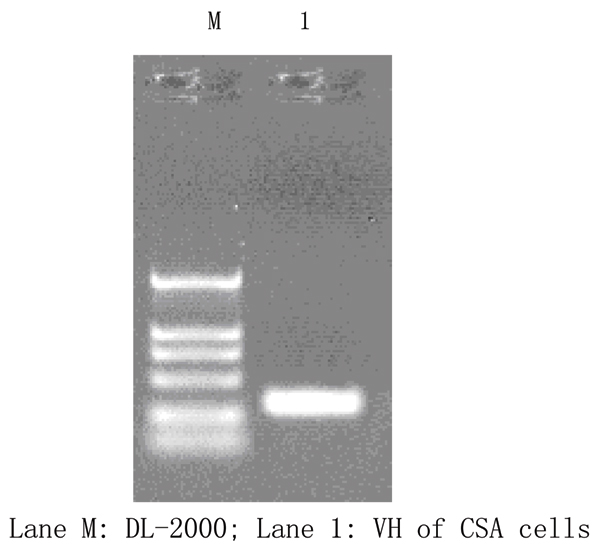
PCR amplification of the VH region of CSA. Lane M: DL-2000; Lane 1: VH of CSA cells.

**Figure 2:**
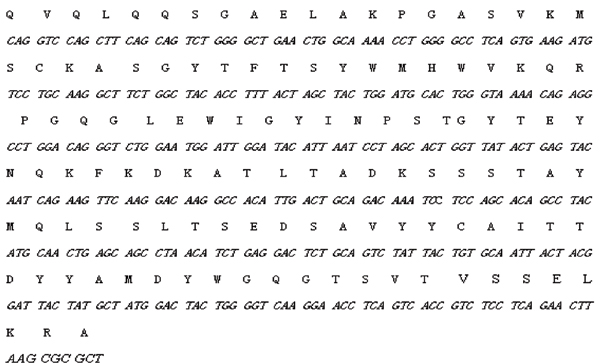
The sequence of the VH region of CSA.

**Figure 3:**
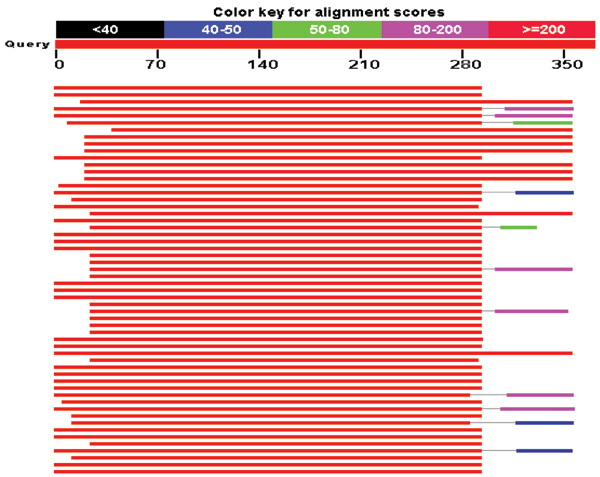
The homology search result provided by NCBI.

(1) In contrast, a good amplification at the expected size was obtained when the novel algorithm was adopted and the 3'RACE and 5'RACE followed with the primer. The VH fragment of the CSA cells was about 399 bp (Fig. 1, Fig. 2).

(2) The result of the homology search using the BLAST algorithm provided by NCBI showed that the VH chain of CSA cell clone was 73% identical and involved in VH7 family (Fig. 3).

## Discussion

### Primer design strategy

Cloning V genes from a number of mouse hybridoma cell lines have been critical for the generation of scFv and the research on the interaction of antibody and antigen. Because 400 bp length of an antibody variable gene has about 10^8 ^variety, amplifying a Fv is more difficult than an unknown gene in other gene families.

In our study, we initially employed the "universal" primers [4] and commercially available mouse primer sets designing for mouse V genes to amplify Fv genes from three hyridoma cell clones. The VL regions of the immunoglobulins cDNA were all amplified successfully. However, the VH region was not amplified from the hybridoma clone CSA. So we had to design our own primers of hybridoma clones.

There are programs which can be used to design primers [5]. However, they have some shortcomings. Firstly, some programs are appropriate for designing primers with small sets of sequences. For example, CODEHOP is a program for designing degenerate primers [6]. CODEHOP works well for small sets of proteins but is inappropriate for constructing primers with very high degeneracy on large sets of sequences. Secondly, some algorithms focus on the coverage of the primers and don't care about the unknown genes. Thirdly, the alignment always focuses on the two ends of the sequences, whereas the most conserved candidates may be in the middle of the related sequences. Some research has noted that there are 20% hybridoma cells clones which can not be amplified successfully with the present programs [7].

Designing degenerate primers manually is appreciated by some people. The Fvs of 100 hybridoma cell lines were amplified successfully by Wang et al. [8]. However, besides being more work than using the programs, this method can not allow a tradeoff between specificity and coverage of the primers [9]. But the successful ratio of Fv amplification can be decreased because of too high specificity or too large coverage of degenerate primers.

To amplify the VH genes of Ig cDNAs from the hybridoma cells, the aims we must achieve are: (1) to align the full length sequences; (2) to design primers of relatively low degeneracy to realize the inherent benefits of a degenerate primer to cover every family sequence; and (3) minimize the number of the specific primers. So we focused on the selection of conserved regions of the sequence and the degeneracy of the primers.

### Algorithm

We have developed a new algorithm for searching for optimal primers to achieve the aims. We prove that the problem of minimizing the number of primers required to amplify a set of DNA sequences is NP-complete. There are two distinct steps. In the first step, all sequences of the variable region from the database were aligned and the conserved region was determined. In the second step, highly degenerate primers in the middle of region of mouse Ig V genes were designed, which is suitable for their PCR amplification. The input of the method is a list of cDNA or DNA sequences and a set of integers that specify the length of the primer.

In general, the conventional protocol for designing the V genes primer is in the leader peptide and in the constant region, or in framework 1 (FR1) and framework 4 (FR4) of the cDNA based on the available sequence data on mouse V segments. For 3' primer design, known constant region sequences are normally chosen as the target sequences. Previous alignment programs often focus on FR1 of the cDNA of the V gene. Afraid of interfering with the antibody function, we abandon selecting the leader peptide as the target for 5'primer design according to the most widespread solutions and selected FR1. Because of the high variety in the end of the Fv is the cure point of defeated amplification, we used two methods of alignment during the alignment in order to find the more conserve region. The first one was all mouse Ig gene sequences listed were aligned within each subgroup defined by Kabat [10]. Based on this alignment, 10 highly degenerate primers at the 5'end of the V FR1 region were designed for VH regions. There were two reasons that we abandoned this approach of alignment. Firstly, our intention was to use as few primers as possible to amplify the target sequence. Secondly, it will not necessarily prevent cross-family amplification if all the primers are used at the same time and nucleotides mismatch may be unnecessarily incorporated into the gene and may interfere with antibody function because of the degenerate nucleotides in primers. The second method was all mouse Ig gene sequences in all subgroups were aligned as one group. So the optimal region, which is in the middle of the VH gene with the most highly conserved sequence, was selected. Only one primer with a few degenerate nucleotides was designed by our program in the end of FR1 region with most highly conserved sequence based on the DNA level or the protein level.

### PCR technique

Traditionally, the alignment of the sequences and designing of primers were based on the end of the target sequences with the currently available programs. Due to the limitations of traditional PCR, the regions in the middle of the sequences were ignored. However, improvement in the technology of PCR has lead to improvement in primer design methods. The number of primer sets designed by our program at the 5' end of the VH region is 10 and less than the number of primers designed by other authors. But we found the most conserved region in the middle of the VH FR1 and a primer with two degenerate nucleotides were designed at this region. The region from part of FR1 to FR4 can be amplified with a Oligo(dT) primer with 3'RACE, because the complete FR1 region can influence the Fv three dimensional structure and the antibody function [11]. The other part of FR1 region was amplified with 5'RACE. So we rescued the complete VH fragment from the immunoglobulin cDNAs using our design program followed by 3'RACE and 5'RACE.

## Conclusion

The program is very effective in sequence alignment. During amplification of an unknown gene, identifying a conserved region is the first and most important step. The lower the variety of sequences is, the lower the difficulty of amplifications is. In our experiment, we found the most conserved region with a heuristic method. The primers designed in this region have higher amplification ability. Then our work became easy and successful.

The program allows a tradeoff between degeneracy and coverage. It is quite effective in designing highly degenerate and highly specific primers for cloning an unknown gene in a large gene family. A primer with a few degenerate nucleotides was designed in the most conserved region in the middle of V region. The target gene was amplified by 3'RACE and 5'RACE. However this was a special case. The program was also quite effective in designing the primers for constructing the antibody library, besides cloning an unknown gene in a large gene family. It was important to note that the design method is a rational combination of computer-aided design and biological experiments.

3'RACE and 5'RACE PCR was a good method for cloning an unknown gene in a large gene family. Since the V region has a high diversity, traditional PCR with degenerate primer sets would produce some mismatch to the template, which would influence the function of the antibody. 3'RACE and 5'RACE can amplify the sequence accurately without any mismatch and assure function on the gene level.

## Methods

The hybridoma cell line CSA against cervical cancer was produced and frozen-preserved in our laboratory. 3'-full RACE and 5'-Full RACE kits were also purchased from Takara Company. The "universal" primers were produced by Takara Company. The commercially available mouse primer sets for mouse Ig gene library construction of recombinant phage antibody system were purchased from Pharmarcia Corporation, U.S.A.

Bioinformatics databases that can be used: NCBI: ; IMGT [12]: .

### Conventional methods

#### 1 RNA isolation and cDNA synthesis

Total cellular RNA was respectively isolated from 5×106 of the hybridoma cells secreting the high specificity and high affinity mAbs using the TRIizol method (Gibco, BRLaithersburg, MD). These were used directly as templates for oligo(dT)-primed cDNA synthesis following a standard procedure in a 20 uL reaction system comprising the following extracted RNA 1 uL, 2 uL 10×reverse transcriptase buffer, 25 mmol/L Mgcl2 8 uL, 10 mmol/L dNTP 2 uL, 5 U/uL AMV 1 uL, 40 U/uL RNA, 2.5 pmol/l Oligo(dT) primer 1 uL. The thermocycling parameters were 10 min at 30°C, 30 min at 50°C, 5 min at 95°C, 5 min at 5°C for 1 cycle.

#### 2 Amplification with "universal" primers

The 5' primers were designed based on Zhou et al. [4] VH1: 5'-SARGTNMAGCTGSAGTC-3' in which S = C or G, M = A or C, R = A or G, and W = A or T; VH2: 5'-SARGTNMAGCTGSAGSAGTCWGG-3'; PCRs were performed in total volumes of 50 uL. Cycling parameters were 94°C for 1 min, 55°C for 1 min and 72°C for 1 s for thirty cycles.

#### 3 Amplification with the primer sets purchased from Pharmacia Company

Reaction volumes were 50 uL with the same PCR parameters as above.

### Novel methods

#### 1 Algorithm

The input of our algorithm is a list of cDNA or DNA sequences. Each sequence is denoted as a string of length *m*, *s*_*i *_= *s*_*i*_[1]*s*_*i*_[2]...*s*_*i *_[*m*], which is over a fixed finite alphabet, i.e. *s*_*i *_[*j*] ∈ Σ = {*A, G, C, T*}, 1≤*i*≤*n*, 1≤*j*≤*m*. All sequences are expressed as a set of string *S *= {*s*_*i*_|1≤*i*≤*n*}. The output is a degenerated string of length *k*, which represents degenerated primers.

In the first step, we align all the input strings and get the conserved regions in them. It is similar to the closest substring problem [13]. Let *s*_*i *_[*j, k*] be a substring of *s*_*i *_= *s*_*i*_[1]*s*_*i*_[2]...*s*_*i*_[*m*] in position *j *and of length *k*, which consists of the sequence of symbols *s*_*i *_[*j*]*s*_*i *_[*j *+ 1]...*s*_*i *_[*j *+ *k *- 1]. We need to find a set of substring *S*[*j, k*] = {*s*_*i *_[*j, k*]|1≤*i*≤*n*]}, which is the most conserved, by minimizing the following objective function.



Where *h*(*a, b*) = |{t|*a*[*t*]≠*b*[*t*]}|, 1≤*t*≤*k *means the hamming distance between string *a *and *b*. *s*^*k *^denotes center string of *S*[*j, k*]. Each letter in the center string is the letter that appears most in same position of *S *[*j, k*]. Let *p*_*i *_= *j *for each *s*_*i*_[*j, k*] denotes the position of the first letter in the substring. The above statement can be formulated as the following optimization problem.



Where *P *= {*p*_*i*_|1≤*i*≤*n*}.

The problem is NP complete, so we need to find an approximation algorithm within polynomial time. The pseudo code is as follows.

Taking 3 strings, *s*_1_, *s*_2_, *s*_3_, randomly from *S *= {*s*_*i*_|1≤*i*≤*n*};

Sampling 3 substrings *s*_1_[*j, k*], *s*_2_[*j, k*]*s*_3_[*j,k*] from *s*_1_, *s*_2_, *s*_3 _respectively;

Finding a substring, which is closest to the center string of the sampled 3 substrings, for every string in *S *= {*s*_*i*_|1≤*i*≤*n*};

Step 1 will be repeated for  times. Step 2 will be repeated for (*m*-*k*+1)^3 ^times. So we get  groups of substrings. Using formulas (1) and (2), the group of substring with the minimum *D *is the most conserved substrings. Step 3 will be repeated for *n *× *k *times. So the whole algorithm will be repeated  times.

Now we get a position set *P *= {*p*_*i*_|1≤*i*≤*n*}. Each element is the beginning position of the conserved region in the corresponding string. In the next step, a degenerated primer is designed in these conserved regions. A PCR primer sequence is called degenerate if some of its positions have several possible bases. The degeneracy of the primer is the number of unique sequence combinations it contains [14]. We overlay all substrings, *s*_*i*_[*p*_*i*_, *k*], as a *n *by *k *matrix. Let *Q *be the set of positions where *s*_*i *_[*j, k*] agree, and *R *= {1,2,...,*k*}-*Q *be the set of positions where *s*_*i*_[*j, k*] disagree. We only need work at the positions, θ, in *R*. A distribution matrix is constructed firstly, which denotes the number of appearances, or count, of each character at each position.

*M*(σ,θ) = |{θ|*s*_*i*_[θ] = σ}|, σ∈Σ, 1≤θ≤|*R*|     (3)

The leading value of column θ, denoted *L*(θ), is defined as the largest value in that column: L(θ) = max{M(σ,θ)|σ∈Σ}. The leading character of column θ is a character *y*(θ), whose count is the leading value: *M*(*y*(θ),θ) = *L*(θ). A column-wise majority string *w *is the string of |*R*| leading characters, one for each column, which is used as initial non-generated string. Then we degenerate the string *w *in order to match a maximum number of strings in the set of *S*_*R *_= {*s*_*i *_[θ ]|1≤*i*≤*n*, 1≤θ≤|*R*|} using minimum degeneracy. The elements except the leading characters in matrix *M*(σ,θ) are sorted from largest to smallest. We select the λ largest elements and degenerate them into their corresponding leading characters. Then a degenerated string w* is obtained. Let *M*(σ_1_, θ_1_)≥ *M*(σ_2_, θ_2_)...≥ *M*(σ_λ_,θ_λ_) denotes the largest λ selected elements and θ* = {θ_1_, θ_2_,..., θ_λ_}, 1≤θ_1_, θ_2_,... θ_λ_≤|*R*| are columns that have selected elements. Let ρ_1 _be the columns that have only one selected element, ρ_2 _be the columns that have two selected elements, ρ_3 _be the columns that have three selected elements, and θ* = ρ_1_⋃ρ_2_⋃ρ_3_. The degeneracy of the string w* is g = . In practice, we don't need to cover all input strings. It is a trade off between degeneracy and coverage (the number of matched input sequences). We can use the parameter λ to adjust this trade off. By combining the characters in positions of *Q *and the characters in positions of *R*, the final primer of length *k *is obtained. There are two parameters in this algorithm, *k *and λ. *k *is the length of the primer, which usually is about 20. The value of λ is determined by degeneracy and depends on the database. The algorithm is implemented on a Pentium IV 2.4 GHz PC with 1 GB DDRAM using Microsoft Visual C++ programming language in WINDOWS_XP environment. A typical execution of this algorithm on 8000 sequences of length 1000 takes approximately 1 minute.

#### 2 primer

The primer designed using the program based on our algorithms is as follows: 5'-AGTGAAGANATCCTGYAAGGG-3'.

#### 3 RACE protocols

3'RACE and 5'RACE were performed following the standard procedure [15,16].

## Authors' contributions

YW drafted most of the manuscript and did the most of the experiments. WC created the tutorial for my experiments. XL conceived of and coordinated the project, drafted parts of the manuscript and created the tutorial. BC constructed the algorithm. All authors read and approved the final manuscript.
